# Green control for inhibiting *Rhizopus oryzae* growth by stress factors in forage grass factory

**DOI:** 10.3389/fmicb.2024.1437799

**Published:** 2024-08-05

**Authors:** Mengdi Dai, Xiangfeng Tan, Xuting Chen, Kangfeng Cai, Yuanxiang Zhong, Ziran Ye, Dedong Kong

**Affiliations:** ^1^Institute of Digital Agriculture, Zhejiang Academy of Agricultural Sciences, Hangzhou, China; ^2^Key Laboratory of Digital Dry Land Crops of Zhejiang Province, Hangzhou, China

**Keywords:** barley, *Rhizopus oryzae*, forage grass factory, stress factor, green control

## Abstract

The forage grass factory could break through the restrictions of land resources, region and climate to achieve efficient production throughout the year by accurate and intelligent management. However, due to its closed environment, mold outbreaks in the forage grass factory were severe, significantly affecting barley production. In this study, 9 contaminated barley tissues were collected and 45 strains were isolated in forage grass factory. After ITS sequencing, 45 strains were all identified as *Rhizopus oryzae*. Through stress factor assays, *R. oryzae* growth was seriously hindered by low concentration of sodium nitrate, high pH value and ozone water treatment. High pH and ozone water affected growth mainly by altering membrane integrity of *R. oryzae*. Sodium nitrate inhibited the growth of *R. oryzae* mainly by affecting the amount of sporulation. Low concentration of sodium nitrate and ozone water did not affect the growth of barley. High concentrations of sodium nitrate (100 mM) and pH values (8–8.5) inhibited barley growth. Among them, ozone water had the most obvious inhibition effect on *R. oryzae*. Large-scale ozone water treatment in the forage grass factory had also played a role in restoring barley production. Taken together, the green techonology to control mold disease and maintain the safety of forage through different physicochemical methods was selected, which was of considerable application value in animal husbandry.

## 1 Introduction

The plant factory mainly realizes precise management through intelligent control of various physical and chemical factors such as temperature, humidity, light, and nutrient supply. The factory system enables the production of vegetables throughout the year and improve the productivity and quality of plants, so as to further solve the problems of shortage or instability of vegetables and food supply, resource efficiency, and environmental impact of agricultural activities (Kozai and Niu, 2016; Kozai, 2018). The forage grass factory adopts the design concept of plant factory. Based on the growth and development characteristics of forage grass, it creates a growth environment suitable for soilless cultivation of forage grass through material exchange and environmental regulation of closed system, so as to achieve the purpose of rapid growth, high quality, high yield, and maximum economic benefit of forage grass. The factory model are especially suitable for arid, semi-arid, non-cultivated land, and alpine areas. The seedlings produced by the forage grass factory have a short production cycle (6–10 d per crop), high planting density, and high yield. At present, forage grass species such as barley, corn, alfalfa, and ryegrass have been produced on a large scale in forage grass factories, but the latter two mainly produce seedling vegetables for human consumption, and do not involve the forage grass function of herbivores (Lee et al., 2010; Fiutak et al., 2019). Among them, barley forage as a kind of high-quality forage grass, has soft and juicy taste, rich nutrition, good palatability of cattle and sheep, rich in vitamins, amino acids and other nutrients, and also contains crude fiber, which is particularly important for maintaining the health of ruminant livestock, and cannot be replaced by grains and other feeds (Fazaeli et al., 2012). Moreover, its digestibility of up to 90% greatly increases the meat production of cattle and sheep (Devendar et al., 2020; Khaziev et al., 2021). It has been used as the main source in forage grass factories for a long time.

In the production process of barley, its performance and nutritional quality are closely related to environmental factors such as cultivation temperature, moisture, air, light, and nutrients (Al-Karaki and Al-Hashimi, 2011; Fazaelil et al., 2011; Naik et al., 2015). The environment with sufficient moisture and warmer cultivation temperature is more suitable for the growth of barley (Podar, 2013). However, such a closed environment is also prone to the growth of microorganisms, especially mold (Grunert et al., 2020). The water and nutrients of the hydroponic system are suitable for the survival and rapid reproduction of microorganisms, and the close proximity and sharing of nutrient solutions will enable the rapid spread of plant diseases (Kanjanamaneesathian, 2015). Due to the large surface and liquid/air boundary, bacteria, and fungi can grow rapidly, causing plant root disease (Dankwa et al., 2020).

Researchers are increasingly realized the implications of microbial development in dairy feed ingredients and feed. The development of mold and other strains in these rations affects not only the nutritional makeup of the food but also its palatability since they produce heat and some mold species produce toxic substances that impair animal health (Mostrom and Jacobsen, 2020). At present, the common pathogens in hydroponic barley include *Pythium, Fusarium, Rhizopus* and *Phytophthora* (Cao et al., 2022). The main means of control are to prevent the growth of pathogens by regularly cleaning and disinfecting equipment, controlling water temperature, using some beneficial bacteria or fungi products, and some chemical products (Dankwa et al., 2020). However, these methods can not completely remove the contamination of pathogens, and at the same time, whether the use of some agents has side effects on herbivores has not been confirmed.

*Rhizopus oryzae* is a well-studied fungus that is frequently used to manufacture some traditional oriental dishes. It is mostly known for being an excellent source of lactic acid (Londoño-Hernández et al., 2017). Additionally, the pathogen is the main cause of mucormycosis, a newly discovered, potentially fatal illness with a growth rate above 50% that is characterized by rapid angioinvasive development (Ma et al., 2009). According to previous reports, *R. oryzae* caused “Rhizopus rot” on a variety of plants, including apple, banana, mulberry, sweet potato, and tomato (Kwon et al., 2012; Wang et al., 2017; Khokhar et al., 2019; Gnanesh et al., 2021). Infection by *R. oryzae* causes the infected site to become soft and decayed. And white mycelium grows around the infected area and had an appearance of cobwebs (Kwon et al., 2015). The biological traits of *R. oryzae* have been the subject of numerous investigations. It can withstand temperatures between 25°C to 45°C, with 38°C being its ideal range (Chen et al., 2021). *R. oryzae* growth is influenced by carbons such ribitol, D-arabitol, and ß-cyclodextrin (Wang et al., 2018), as well as pH (Chen et al., 2021).

In our study, we first repoted that *R. oryzae* infected the roots of barley in forage grass factories and reduced barley yield. Through a variety of physical and chemical methods, a green control was summarized to inhibit the growth of *R. oryzae* in plant factories without affecting barley development.

## 2 Materials and methods

### 2.1 The growth condition of plant materials

The barley cultivars (*Hordeum vulgare* L.) were grown in an artificial climate chamber and forage grass factory. In artificial climate chamber, barley were grown in culture bottles. And the climate chamber was composed of three microclimate chambers and a management system that controlled the temperature, relative humidity, and LED illumination. Its dimensions were 1,130 mm (L), 795 mm (W), and 1,920 mm (H). The air temperature was set as 25 ± 2°C with a relative humidity of 85%. The forage grass factory is a 20 inch [6,000 mm (L)^*^2,400 mm (W)^*^2,450 mm(H)] container placed in Infite Industrial Park, Hangzhou City, China. The factory was equipped with an environmental control system to control temperature, humidity and carbon dioxide; liquid circulation system to achieve 6–8 times a day spray; automatic control equipment to adjust temperature and humidity, carbon dioxide concentration, and liquid pH value in real time. At the same time, the factory was equipped with two sets of three-layer planting racks [4,000 mm (L)^*^460 (W) mm^*^2,190 mm (H)] and 24 plant growth lights, which could achieve multiple rounds of barleys. The air temperature was set as 25 ± 2°C with a relative humidity of 88%, carbon dioxide concentration of 480 ± 10 ppm in the forage grass factory.

### 2.2 Isolation and cultivation of strains

The infected barley plants in the forage grass factory were randomly selected and isolated by conventional tissue isolation method (Lozada, 1995). Briefly, the roots of infected barley plants were cut, and tissues of 5 mm × 5 mm were cut at the junction of diseased and healthy, immersed in 75% alcohol solution for disinfection for 10 s, then put into 3% sodium hypochlorite solution for disinfection for 45 s, washed with sterilized water for 3 times, and cultured on potato dextrose agar (PDA) medium under dark conditions at 25°C. The mycelia on the edge of the colony were selected and transferred to a new PDA plate for purification, and then placed on the PDA slope at 4°C for store.

### 2.3 Total DNA extraction and identification

Genomic DNA was extracted according to the DNA extraction kit (Tiangen, Shanghai). The internal transcribed spacer (ITS) genes was amplified for identification (Zhu et al., 2022). Primers were listed in Supplementary Table S1. PCR amplification was conducted as described by the method of Zhang et al. (2011). PCR products were sequenced using ABI3730 (Tsingke, Beijing), and the sequencing results were compared with the BLAST sequence. Mega version X was used for the construction of phylogenetic trees.

### 2.4 Stress assay on *R. oryzae*

The identified fungi *R oryzae* strain was cultured on potato dextrose agar (PDA, Difco, Detroit, MI, USA) at 25°C in the dark. Fresh hyphae plug was picked up from the edge of *R. oryzae* cultured for 2 d with a diameter of 5 mm stopperborer, and inoculated on the 7 cm medium with different conditions. Under dark conditions, the strain was cultured at 25°C for 1 day, and the colony diameter was measured. The stress medium is as follows (Table 1):

**Table 1:** Different stress assay on *R. oryzae*.

**Stress factor**	**Different treatment**
Carbon sources	Glucose, maltose, fructose, sucrose, mannose, and lactose
pH stress	pH = 4.5, 5, 5.5, 6, 6.5, 7, 7.5, 8, 8.5
Nitrate stress	0.1 mM, 1 mM, 10 mM, and 100 mM
Ozone water stress	2 ppm, 1 ppm, 0.5 ppm
Salt stress	0.2 M sodium chloride, 0.3 M sodium chloride, 0.2 M potassium chloride, and 0.3 M potassium chloride
Phosphorus stress	5 mM lecithin, 50 mM lecithin, 5 mM potassium dihydrogen phosphate, and 50 mM potassium dihydrogen phosphate

For different carbon sources stress assay, using the basic medium MM (1% glucose) as blank control, the contents of glucose in MM medium were replaced with maltose, fructose, sucrose, mannose, and lactose, respectively.

For different pH stress assay, the pH value of MM medium were adjusted by hydrochloric acid and sodium hydroxide respectively.

For stress assays with different concentrations of nitrate, the concentrations of sodium nitrate in MM medium were replaced with 0.1 mM, 1 mM, 10 mM, and 100 mM concentrations.

For stress assays with different concentrations of ozone water stress, add 5 mL ozone water (the initial concentration is 10 ppm) to 20 mL MM medium (2 ppm), 2.5 mL ozone water to 22.5 mL MM medium (1 ppm), and 500 μL ozone water to 25 mL medium (0.5 ppm), and pour it into the culture dishes.

For salt stress assays, 0.2 M sodium chloride, 0.3 M sodium chloride, 0.2 M potassium chloride, and 0.3 M potassium chloride were added to PDA medium, respectively.

For stress assays of different phosphorus, 5 mM lecithin, 50 mM lecithin, 5 mM potassium dihydrogen phosphate, and 50 mM potassium dihydrogen phosphate were added to MM medium, respectively.

### 2.5 Phenotypic characterization of *R. oryzae* under stress

The strain was cultured on 7 cm culture dish containing 17.5 mL MM medium and stress factors (carbon sources stress, pH stress, different concentrations of nitrate stress, different concentrations of ozone water stress, salt stress, and different phosphorus stress) at 25°C in dark for 1 day. Fifty photos were taken for each treatments and subsequent statistical analysis was performed.

#### 2.5.1 Effect on sporification

To measure sporulation and sporangium, 3 ml sterile water were added into the MM medium. The aerial hypha were scraped on the medium with a spreader, and 15 μl fluid was absorbed to observe the sporangium production under a light microscope. The remaining fluid was filtered with three layers of filter paper, and the filtrate was centrifuged and 100 μl sterile water was added to precipitate. The sporulation and sporangium were counted with a hemocytometer under light microscope.

#### 2.5.2 Effect on mycelium reduction

To measure mycelium weight, different stress factors were added to MM liquid medium, and conventional MM medium was used as the control. Five fungal plugs (5 mm) were picked up by a stopperborer and inoculated in the liquid medium, The medium with fungal plugs were shaked at 25°C and 150 rpm/min for 5 d. The mycelia was filtered with three layers of sterile filter paper, and the filtered mycelia was dried with absorbent paper. The mycelium were weighed the fresh weight.

#### 2.5.3 Effect on the integrity of the cell membrane

To measure the integrity of fungal mycelia membrane, mycelia cultured in MM liquid medium was rinsed 3 times with PBS buffer. 0.5 g mycelia was weighed and placed in a 50 mL centrifuge tube, and different stress factors were added respectively. After 2 h treatment, the solution was centrifuged at 5,000 rpm/min, and the solution without mycelia at the same concentration was used as a negative control. The absorption value of supernatant at 260 nm was detected.

### 2.6 Barley stress assay and co-culture

In artificial climate chamber, barley seeds were ground to remove capsules, then surface-sterilized in 75% ethanol for 5 mins and in 1.0% sodium hypochlorite solution (5% active chlorine) for 10 mins. The seeds' surface was then rinsed with sterile water. Ten sterilized seeds were planted in the 1/2 MS solid medium in a 9 cm culture dish. After 3–4 d, 8 seedlings were transferred into a sealed tissue culture bottle (the bottom diameter is 95 mm, the height is 185 mm, the capacity is 1,000 ml), which contained 1/2 MS solid medium. For stress assay, the 1/2 MS medium was supplemented stress factors as described above. For co-culture, each culture bottle was planted barley seedlings with three fungal plugs of *R. oryzae*. Each treatment had three tissue culture bottles, and each experiment was repeated three times. In forage grass factory, the barley seeds were rinsed in water to remove dented grains, and then soaked in sodium hypochlorite solution for 2 h. Then 50 g seeds were evenly scattered in the plastic square dish with holes in the bottom [135 mm (L)^*^83 mm (W)^*^53 mm (H)] and placed in the container for planting. After 4 d, the stress solution was prepared, and equal amounts were poured into the barley seedling tray for stress treatment. The stress treatment was carried out once a day until harvest.

### 2.7 Phenotype measurement on barley

After 7 d planting, phenotype measurement was conducted at a fixed time point. Five barley seedlings were randomly selected for each tissue culture bottle to measure the plant height. The plant height was the linear distance between the highest point of the canopy leaf and the medium. Since there is only 8 barleys in the tissue culture bottle and the weight is relatively light, the fresh weight is mainly determined in forage grass factory. The above-ground part of barley seedlings was cut and the fresh weight was measured.

### 2.8 Determination of physiological indexes

Plant leaves that were fully developed and in good health were chosen at random from each treatment for analysis. Three repeats of the study were conducted for every physiological index. The crude fat content of plants was determined by Soxhlet extraction method. The total starch content of plants was measured by acid hydrolysis method-DNS. The content of crude protein was determined by using sulfuric acid-catalyst digestion and Kjeldahl nitrogen determination method.

### 2.9 Statistical analysis

One-way ANOVA and independent-samples *t*-test were used to statistically analyze the data using SPSS (version 16.0, IBM, Chicago, IL, USA) software. The values demonstrated the mean ± standard error.

## 3 Results

### 3.1 Discovery and identification of mold in forage grass factory

**Figure 1:**
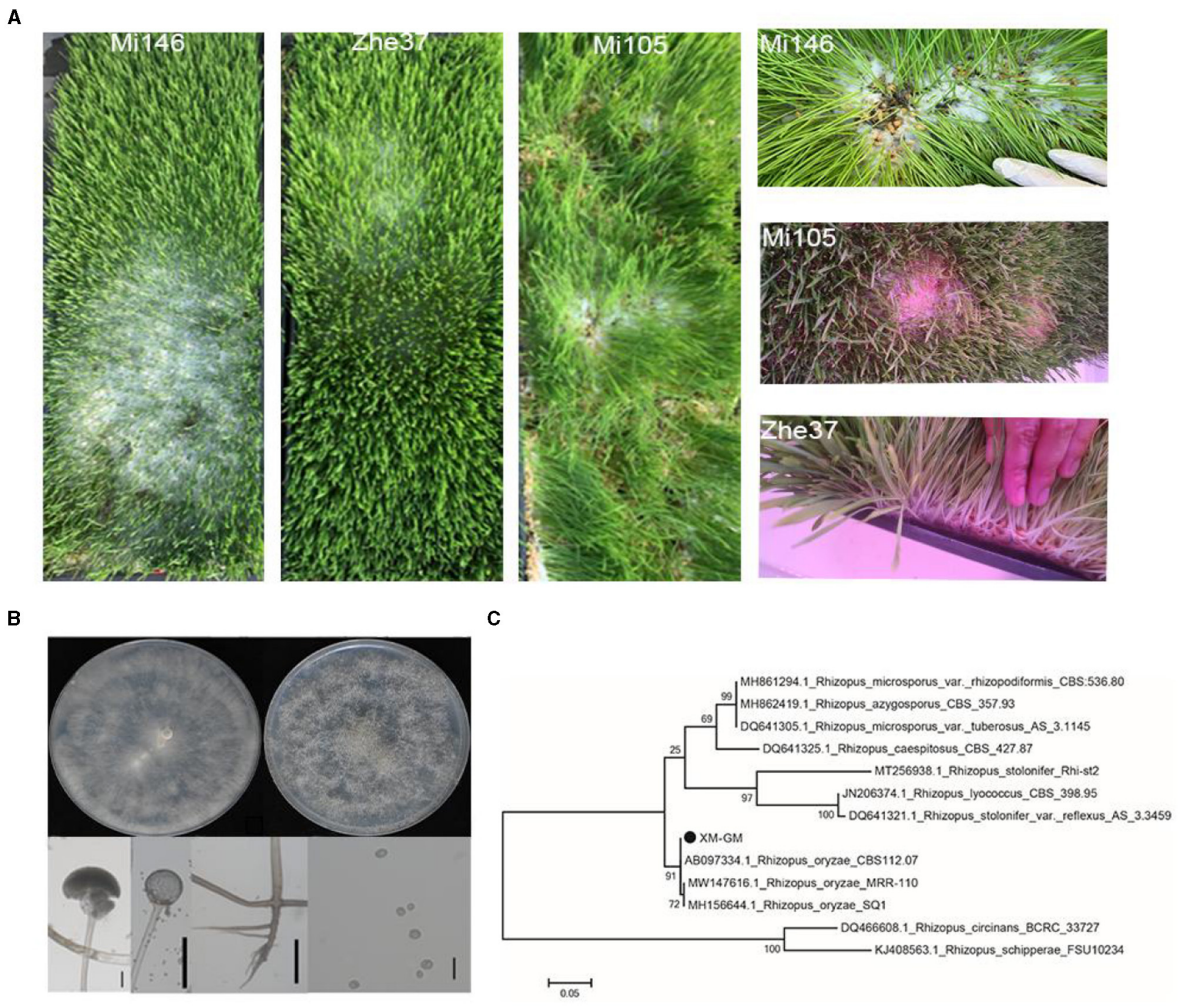
Identification and morphological characteristics of mold in forage grass factory. **(A)** Mold infected barley varieties Mi146, Zhe37, and Mi105 on planting trays at 7 d. **(A)** Mycelium morphology of isolated strain at 2 d of growth. **(B)** The topper lines from left to right were Mycelium morphology of isolated strain at 2 d of growth and 4 d, respectively. At 2 d, the mycelium was generally grayish white, and there were no black sporangia spores on the top. At 4 d, many black sporangia were produced. The lower lines from left to right were sporangium, sporangiophore, rhizoid, and sporangiospores of isolated strain, respectively. Bar = 20 μm. **(C)** Phylogenetic tree analysis based on ITS sequence. The isolated strain XM-GM was identified as *R. oryzae*. The evolutionary history was inferred using the Neighbor-Joining method. The base substitution sites were shown by the bar at 0.04.

We planted 9 different varieties of barley seedlings in the forage grass factory. After growing under the same environmental control condition for 7 d, we had a phenotypic observation and found that three barley varieties Mi146, Mi105, and Zhe37 had serious mold outbreaks. There is a large amount of white mycelium in the roots of barley, and the mycelium has black spots on the top. In severe cases, the white mycelium extends to the barley leaves (Figure 1A). We randomly selected ninety contaminated barley tissues to isolate the contaminated fungi and obtained 45 isolated strains. The morphological characteristics of the contaminated fungi were observed on the PDA medium. The results showed that the morphological characteristics of the 45 strains were the same species. The colony was initially white, then many black sporangia were produced. The sporangium was spherical or sub-spherical, with a diameter of 72.37 ± 4.21 μm (*n* = 50). The rhizoid was dark brown and 4.37 ± 1.05 μm wide (*n* = 50). The sporangiospores were non-septate, globose or sub-globose, and about 4.35 ± 0.74 μm in diameter (*n* = 50) (Figure 1B). Five typical strains were selected for ITS sequence determination. By phylogenetic tree analysis, the isolated strain XM-GM and *Rhizopus oryzae* clustered into a clade, and the clade support was 91% (Figure 1C). In summary, the morphological characteristics of the barley seedling contaminated were observed, ITS sequence was determined, and phylogenetic tree analysis of ITS sequence was performed. The results showed that the contaminated fungi of barley seedling was *Rhizopus oryzae* of Rhizopus.

### 3.2 Impact of stress factors on *Rhizopus oryzae*

**Figure 2:**
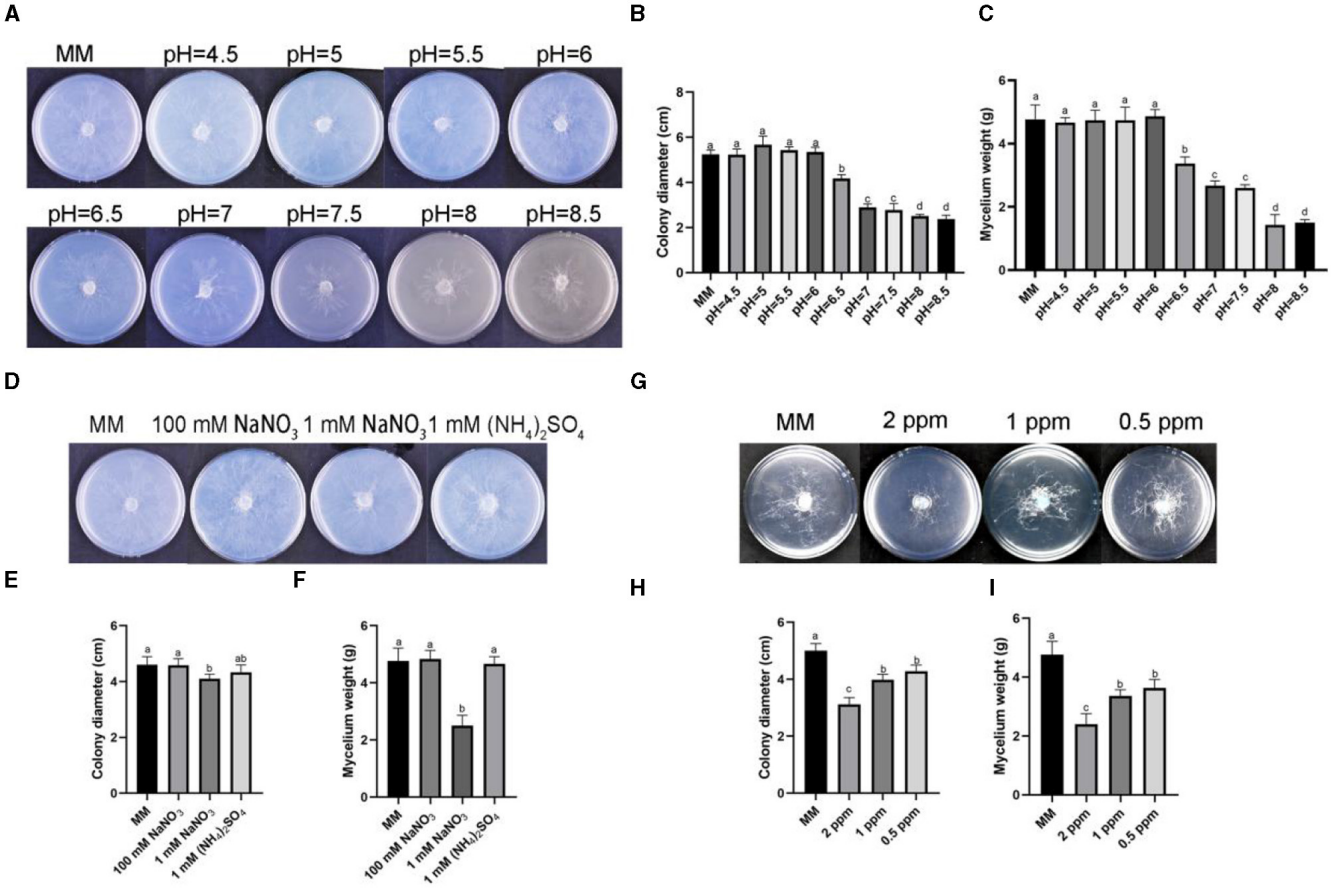
Stress assays on *R. oryzae*. **(A)** The strain cultured on MM media with different pH under dark conditions for 1 d. **(B)** The colony diameter on MM media with different pH. **(C)** The mycelium weight on MM media with different pH. **(D)** The strain cultured on MM media containing 100 mM NaNO_3_, 1 mM NaNO_3_, and 1 mM (NH_4_)_2_SO_4_ under dark conditions for 1 d. **(E)** The colony diameter on MM media containing 100 mM NaNO_3_, 1 mM NaNO_3_, and 1 mM (NH_4_)_2_SO_4_. **(F)** The mycelium weight on MM media containing 100 mM NaNO_3_, 1 mM NaNO_3_, and 1 mM (NH_4_)_2_SO_4_. **(G)** The strain cultured on MM media containing 2 ppm ozone water, 1 ppm ozone water, and 0.5 ppm ozone water under dark conditions for 1 d. **(H)** The colony diameter on MM media containing 2 ppm ozone water, 1 ppm ozone water, and 0.5 ppm ozone water. **(I)** The mycelium weight on MM media containing 2 ppm ozone water, 1 ppm ozone water, and 0.5 ppm ozone water. Error bars mean standard deviation from three replicate samples. Different letters represented significant differences (*p* < 0.05).

To observe the growth of *R. oryzae* under different stresses, we conducted plate experiments on the response of *R. oryzae* to stress factors, and found out the stress factors affecting the growth of *R. oryzae*. The results showed that the growth of *R. oryzae* was inhibited under alkaline condition (pH = 7–8.5) (Figure 2A). Mycelium weight and colony diameter decreased significantly when pH = 7–8.5 compared with acidic environment (Figures 2B, C). Under different nitrogen source conditions, it was found that *R. oryzae* was more able to utilize organic nitrogen than inorganic nitrogen source (Figure 2D). The colony diameter and fungal biomass of *R. oryzae* grew under 0.1 mM sodium nitrate stress were significantly lower than other nitrogen conditions (Figures 2E, F). Under different concentrations of ozone water, the growth of *R. oryzae* was significantly inhibited (Figure 2G). And the inhibition effect gradually decreased with the decrease of concentrations (Figures 2H, I). Under different carbon sources, *R. oryzae* could grow normally (Supplementary Figures S1A, B). Different concentration of salt stress factors and phosphorus culture conditions could promote the growth of *R. oryzae* (Supplementary Figures S1C–F).

To observe whether green biocontrol fungi could inhibit the growth of *R. oryzae*, the experiment was conducted between *R. oryzae* and *Piriformospora indica* and *Bacillus subtilis*. The diameter of *R. oryzae* was not significantly different from that of the control, indicating that green biocontrol fungi could not directly inhibit the growth of *R. oryzae* and did not secrete any antagonistic substances against *R. oryzae* (Supplementary Figure S2).

### 3.3 Mechanism of stress factors on *R. oryzae*

**Figure 3:**
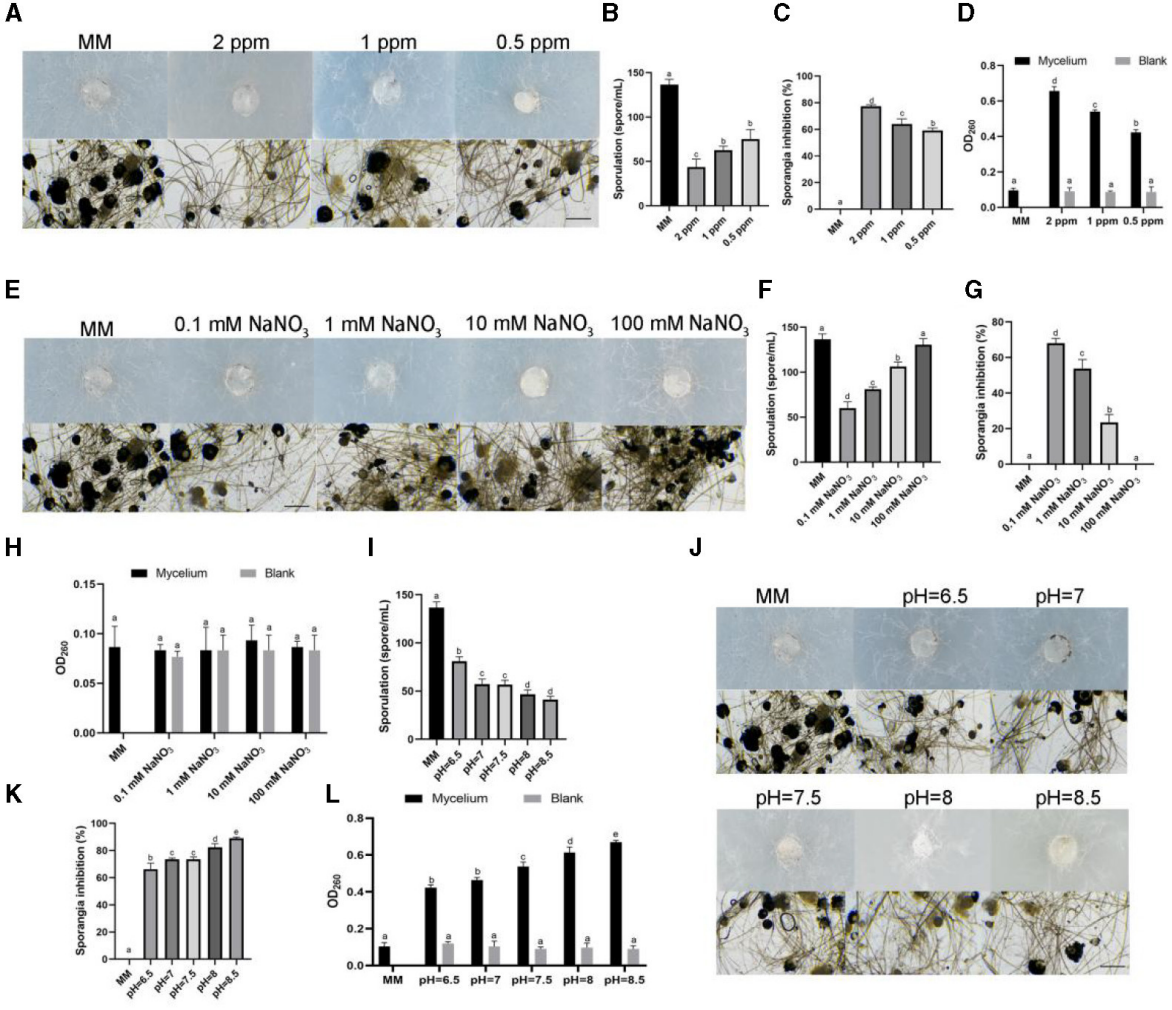
Effects of different stress factors on sporangium, sporulation and membrane integrity of *R. oryzae*. **(A)** The aerial hyphae and sporangium morphology of *R. oryzae* growing under 2 ppm ozone water, 1 ppm ozone water, and 0.5 ppm ozone water. Bar = 10 μm. **(B)** The spotulation of *R. oryzae* growing under 2 ppm ozone water, 1 ppm ozone water, and 0.5 ppm ozone water. **(C)** The sporangium inhibition of *R. oryzae* growing under 2 ppm ozone water, 1 ppm ozone water, and 0.5 ppm ozone water. **(D)** The membrane integrity of *R. oryzae* growing under 2 ppm ozone water, 1 ppm ozone water, and 0.5 ppm ozone water. **(E)** The aerial hyphae and sporangium morphology of *R. oryzae* growing under 0.1 mM NaNO_3_, 1 mM NaNO_3_, 10 mM NaNO_3_, and 100 mM NaNO_3_. Bar = 10 μm. **(F)** The spotulation of *R. oryzae* growing under 0.1 mM NaNO_3_, 1 mM NaNO_3_, 10 mM NaNO_3_, and 100 mM NaNO_3_. **(G)** The sporangium inhibition of *R. oryzae* growing under 0.1 mM NaNO_3_, 1 mM NaNO_3_, 10 mM NaNO_3_, and 100 mM NaNO_3_. **(H)** The membrane integrity of *R. oryzae* growing under 0.1 mM NaNO_3_, 1 mM NaNO_3_, 10 mM NaNO_3_, and 100 mM NaNO_3_. **(I)** The aerial hyphae and sporangium morphology of *R. oryzae* growing under different pH. Bar = 10 μm. **(J)** The spotulation of *R. oryzae* growing under different pH. **(K)** The sporangium inhibition of *R. oryzae* growing under different pH. **(L)** The membrane integrity of *R. oryzae* growing under different pH. Error bars mean standard deviation from three replicate samples. Different letters represented significant differences (*p* < 0.05).

To clarify the mechanism of different stress conditions affecting the growth of *R. oryzae*, we observed the aerial hyphae and sporangium of *R. oryzae* growing on stress medium. The results showed that the aerial hyphae of *R. oryzae* growing under ozonated water stress was thinner than that of the control (Figure 3A). It was found that ozone water could inhibit sporospore production. In 2 ppm ozone water treatment, its sporulation was only 1/3 of the control (Figure 3B). With the decrease of concentrations, the spore production also increased. Through observation of sporangium, it was found that *R. oryzae* grown on MM medium produced a large number of sporangium, while the number of sporangium of *R. oryzae* was significantly reduced under ozone water treatment, and the sporangium inhibition rate reached 77.38% under 2 ppm ozone water treatment (Figure 3C). When the concentrations decreased, the sporangium inhibition rate decreased, indicating that ozone water could significantly inhibit the production of sporangium in *R. oryzae*. By measuring the integrity of mycelium membrane, it was found that the absorption value of the solution was increased by the treatment of ozone water, indicating the leakage of nucleic acid. However, the absorption value of ozone water without adding mycelium did not change with the change of concentrations, suggesting that this phenomenon was not caused by the presence of ozone water in the solution, but by the change of membrane integrity of *R. oryzae*. The results showed that ozone water could destroy the membrane structure of *R. oryzae* and cause the change of membrane integrity (Figure 3D).

Besides, we also observed the growth condition of *R. oryzae* under different pH and different concentrations of sodium nitrate stress (Figures 3E, J). The results showed that different concentrations of nitrate also affected the sporulation and sporangium generation of *R. oryzae*, and with the increase of nitrate concentration, sporangium production gradually increased, and sporangium inhibition rate gradually decreased (Figures 3F, G). However, the increase of nitrate concentration did not affect the change of absorption value in *R. oryzae* solution, indicating that the change of nitrate concentration did not affect the integrity of mycelia (Figure 3H). In addition, pH affected sporulation, sporangium generation and mycelium integrity of *R. oryzae*, thus inhibiting the growth of *R. oryzae*. And this inhibition gradually increased with the increase of pH value (Figures 3I, K, L).

### 3.4 Effects of stress factors on the barley-*R. oryzae* interaction relationship

**Figure 4:**
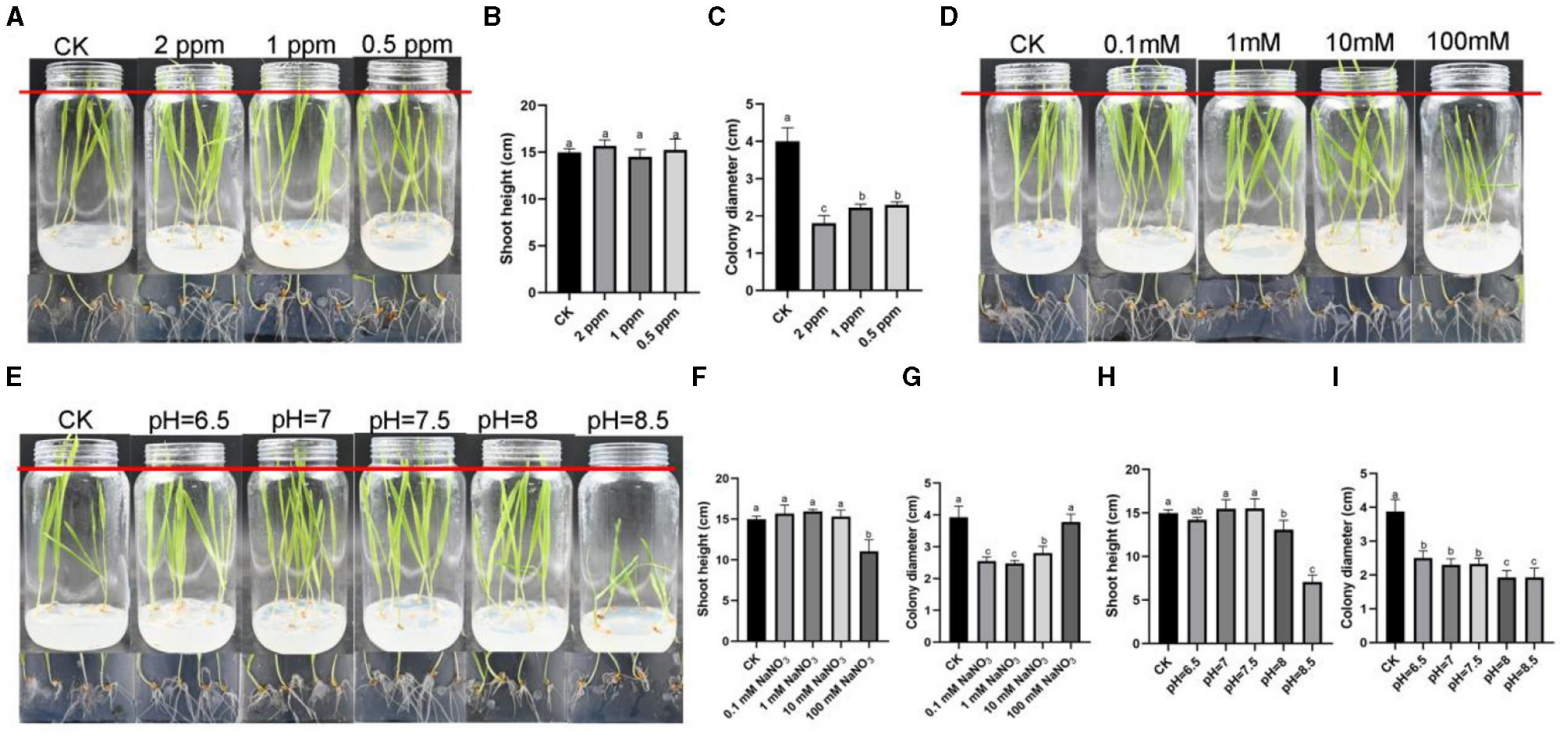
The phenotype of barley-*R. oryzae* interaction relationship under different stresses. **(A)** The phenotypes of barley and *R. oryzae* grown for 7 d under 2 ppm ozone water, 1 ppm ozone water, and 0.5 ppm ozone water. **(B)** The shoot height of barley grown for 7 d under 2 ppm ozone water, 1 ppm ozone water, and 0.5 ppm ozone water with *R. oryzae* presence. **(C)** The colony diameter of *R. oryzae* on 1/2 MS medium containing 2 ppm ozone water, 1 ppm ozone water, and 0.5 ppm ozone water. **(D)** The phenotypes of barley and *R. oryzae* grown for 7 d under 0.1 mM NaNO_3_, 1 mM NaNO_3_, 10 mM NaNO_3_, and 100 mM NaNO_3_. **(E)** The phenotypes of barley and *R. oryzae* grown for 7 d under different pH. **(F)** The shoot height of barley grown for 7 d under 0.1 mM NaNO_3_, 1 mM NaNO_3_, 10 mM NaNO_3_, and 100 mM NaNO_3_ with *R. oryzae* presence. **(G)** The colony diameter of *R. oryzae* on 1/2 MS medium containing 0.1 mM NaNO_3_, 1 mM NaNO_3_, 10 mM NaNO_3_, and 100 mM NaNO_3_. **(H)** The shoot height of barley grown for 7 d under different pH with *R. oryzae* presence. **(I)** The colony diameter of *R. oryzae* on 1/2 MS medium with different pH. Error bars mean standard deviation from three replicate samples. Different letters represented significant differences (*p* < 0.05).

In order to test whether these three stress factors could inhibit the growth of *R. oryzae* without affecting the growth of barley, we conducted co-culture assay between barley and *R. oryzae* under the above stress conditions (Figures 4A, D, E). The results showed that, consistent with the results of the previous barley tissue culture assays (Supplementary Figure S3), except for the conditions of 100 mM sodium nitrate, pH = 8 and pH = 8.5, the barley growing under other conditions could grow normally compared with the control, indicating that the influence of *R. oryzae* on the growth of barley was less than that under stress conditions (Figures 4B, F, H). In different concentrations of ozone water treatment, 2 ppm inhibition of *R. oryzae* was the best, and the colony diameter was 1.8 cm, which was much smaller than that of the control (4 cm) (Figure 4C). In different concentrations of sodium nitrate treatment, 0.1 mM sodium nitrate (2.55 cm) and 1 mM sodium nitrate (2.475 cm) could achieve better inhibition effect on *R. oryzae* (Figure 4G). Under different pH treatments, the inhibition effect on *R. oryzae* was obvious when pH = 8 and 8.5, but the growth of barley was poor (Figure 4I). In summary, considering the inhibition effect and operability, we thought that 2 ppm ozone water was more suitable for controlling *R. oryzae* in forage grass factories.

### 3.5 Application of ozone water in forage grass factory

**Figure 5:**
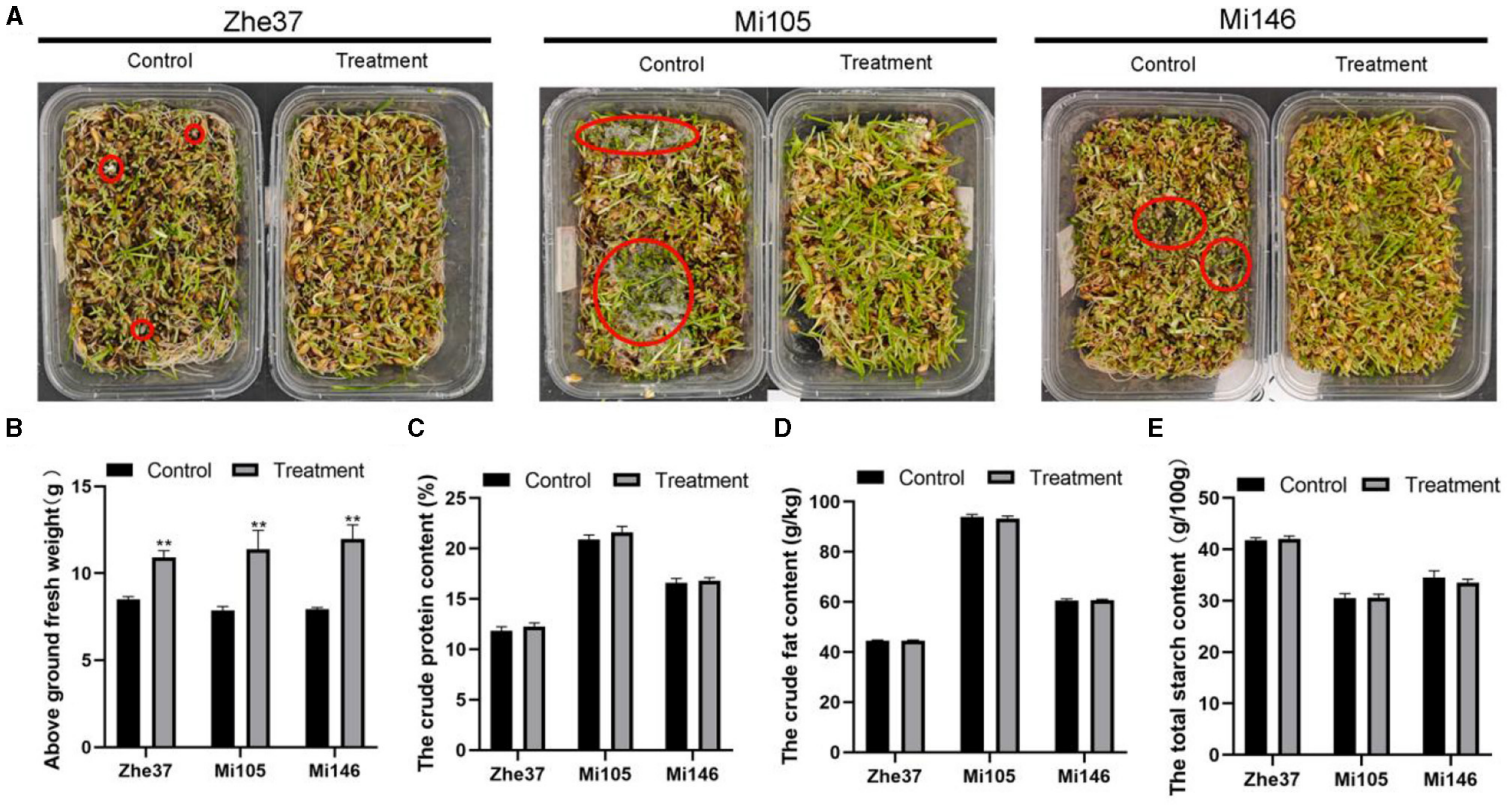
Control effect of ozone water in forage grass factory. **(A)** Mold outbreaks in forage grass factory after 2 ppm ozone water treatment. The barley varities include Zhe37, Mi105, and Mi146. The red circle represents the mold growth point. **p < 0.01. **(B)** The above ground fresh weights of Zhe37, Mi105, and Mi146 barley varities treated with 2 ppm ozone water in forage grass factory. **(C)** The crude protein content of Zhe37, Mi105, and Mi146 barley varities treated with 2 ppm ozone water in forage grass factory. **(D)** The crude fat content of Zhe37, Mi105, and Mi146 barley varities treated with 2 ppm ozone water in forage grass factory. **(E)** The total starch content of Zhe37, Mi105, and Mi146 barley varities treated with 2 ppm ozone water in forage grass factory. Error bars mean standard deviation from three replicate samples.

To verify whether ozone water could effectively inhibit the outbreak of *R. oryzae* in forage grass factory, we planted three barley varieties including Mi105, Zhe37, and Mi146 which were susceptible to *R. oryzae*. It could be seen from the results that the mold in the root of barley was controlled to a certain extent by spraying 2 ppm ozone water daily (Figure 5A), and the above ground of fresh weight was also increased compared with the control. The fresh weight of barley variety Mi105 was 11.39 g, which increased by 45% compared with untreated (Figure 5B). Barley quality indexes including crude fat content, crude protein content, and total starch content were determined. It was found that the barley quality was not affected by 2 ppm ozone water treatment, and there was no significant difference compared with the control (Figures 5C–E).

## 4 Discussion

Mold contamination causes huge economic losses to food industry (Dantigny et al., 2005). These losses are difficult to assess because they fluctuate with location, season, and type of spoiled product. 131 notifications about the presence of mold in various animal and vegetable food products as well as dietary supplements were approved between 2018 and 2022 in the European Union (RASFF).^1^ And the highest incidence of mold in these products was in cereal and bakery products. In our study, we also found mold outbreak in forage grass factory. And the contamination caused the decrease of barley yeild (Figure 1A). By ITS sequencing, we identified *R. oryzae* as a main contaminator in the forage grass factory (Figures 1B, C). The interaction between *R. oryzae* and barley didn't report in other study. The main reason for this interaction might be due to the special environment in plant factory. Different from the natural environment outside, the humidity inside the plant factory is high, the temperature changes are small, the air circulation is slow, and it is easier to form an acidic environment. Such environmental conditions promoted *R. oryzae* to become the dominant colony, interact with barley, and affect the yield. Therefore, plant factory as a special habitat environment, its internal microbial dynamic changes, microbial- plant interaction forms, deserves our further study.

Nitrogen, as an essential element for biological growth and development, is supplemented in plant factories mainly by nutrient solution (Vidal et al., 2020). Different organisms have different preferences for different concentrations and forms of nitrogen (Marschner, 1995; Chen et al., 2004). In recent study, it was found that around 25 nitrogen sources (amino acids) significantly inhibited the growth of *R. oryzae* (Li et al., 2023). In this study, it was found that *R. oryzae* prefered nitrates for growth (Figure 2D), indicating that *R. oryzae* might have the ability of denitrification. As a pathway for protein synthesis, nitrogen also closely related to the growth of mycelia (Morschhäuser, 2011). From the results, we also found that when nitrate nitrogen was reduced, the sporulation and biomass of *R. oryzae* were reduced (Figure 3E). Thus, by controlling the concentration of nitrate, we could effectively inhibit the growth of *R. oryzae* and controled the outbreak of *R. oryzae* from the perspective of nitrogen source utilization. Moreover, it is important for plants to maintain appropriate nitrate concentrations. From our results, we found that when the concentration of sodium nitrate in the environment reached 100 mM, barley growth was hindered. When sodium nitrate concentration decreased, barley grew normally. In the barley-*R. oryzae* symbiosis system, the barley grew normally under the condition of inhibiting *R. oryzae* growth only by maintaining the appropriate sodium nitrate concentration (0.1 mM and 1 mM).

Besides, pH is also a limiting factor for fungal-plant interaction. The pH value suitable for mold growth varies with different species. The optimal pH of common *Penicillium, Aspergillus, Streptomyces* and other strains is about pH 5–6. The low pH preference of some molds that cause fruit decay is likely an adaptation to the growth of acidic host fruits (Buijs et al., 2021). In the forage grass factory, a large number of barley seeds were laid on the planting tray. The too-dense seeds planting made the barley undergo anaerobic respiration and generate acetic acid, resulting in a decrease in environmental pH. Lower pH was suitable for the growth of molds such as *R. oryzae* (Figures 1A, 2A). *R. oryzae* is mainly used in fermentation industry and is suitable for growing in acidic environment (Sebastian et al., 2021). From the result, it was also inferred that when the pH value exceeded 6.5, the growth of *R. oryzae* was significantly limited (Figure 2A), which might be related to the alkaline condition disrupting membrane integrity (Figure 3L). Similarly, barley prefers to grow in slightly acidic and neutral environment (pH value between 6–7.8). So even though alkaline conditions can inhibit the outbreak of *R. oryzae*, they will also inhibit the growth and development of barley. When the pH value of the environment was 7–7.5, the expansion of *R. oryzae* could be better inhibited than control without affecting the growth of barley (Figure 4E). Therefore, the outbreak of *R. oryzae* could be inhibited by adjusting the pH value of nutritional solution properly during the subsequent cultivation of barley in forage grass factory.

Ozone has a potent microbicidal action against bacteria, fungi, parasites, and viruses when they are present in low-ozone-demand media. As a safe antibacterial agent, the FDA authorized ozone for the food industry about 20 years ago (Asokapandian et al., 2018). Ozone can be used as an aqueous or gaseous substance. The benefits of ozone over other chemical oxidants are as follows: (a) it can be produced on-site; (b) it can be administered in both gaseous and aqueous forms; (c) it doesn't leave any residue after contact; (d) it can be produced in large quantities; and (e) it doesn't have any hazardous disposal requirements (Pandiselvam et al., 2019; Torres et al., 2019). Ozone has the ability to deactivate a variety of microorganisms, including viruses and protozoa (Brié et al., 2018), pathogenic bacteria like *Salmonella typhimurium, Escherichia coli*, and *Listeria monocytogenes* (Alwi and Ali, 2014; Song et al., 2015), and fungi (Allen et al., 2003; Watanabe et al., 2010). The ozone's inactivation strategies against bacteria include ozone penetrating cells, targeting components of cell membranes, inactivating enzymes, and breaking down gDNA and total RNA's genetic materials. These activities finally lead to cell lysis and the leaking of cellular contents. In our study, we produced ozone water to control *R. oryzae*, and found that it had the best inhibitory effect on *R. oryzae* without affecting barley growth (Figures 4A–C). And the main reason was by changing the integrity of its cell membrane, resulting in the leakage of cellular substances (Lee and Kim, 2022) (Figure 3D). This treatment does not affect the normal growth of the barley due to its ability to self-decomposition, rapid action, and strong oxidative properties to produce oxygen (Sarron et al., 2021). By using ozone water in the forage grass factory, it was found that it suppressed the outbreak of *R. oryzae* and restored barley production (Figure 5). In addition, unlike the other two influencing factors that require changing the composition of the nutrient solution, ozone water can enter the grass factory through additional addition, which is more operational. Therefore, ozone water is more suitable for the application of ozone (whether in liquid or gas form). Ozone will be a promising industrial fungistatic technology and has gained great interest in the food industry.

## 5 Conclusion

Based on the controllable environment of forage grass factory, this study explored the influence of different stress factors on the growth of mold and barley. The fungi were identified by ITS sequencing and the dominant fungus *R. oryzae* was isolated. By exploring the effect of different stress factors on the prevention and control of mold disease and the safety of barley, the green-technology of the prevention and control of mold disease in forage grass factory was studied. We found out the best means of controlling *R. oryzae*, that is, by ozone water to inhibit the growth, while not affecting the growth of barley. The molecular mechanism of ozone water treatment is to change the membrane integrity of *R. oryzae*. It was also used in the forage grass factory and obtained a significant prevention effect. These results will play an important role in the promotion of industrial forage grass culture technology and the development of animal husbandry.

## Data availability statement

The datasets presented in this study can be found in online repositories. The names of the repository/repositories and accession number(s) can be found in the article/Supplementary material.

## Author contributions

MD: Investigation, Writing – original draft. XT: Methodology, Writing – review & editing. XC: Investigation, Methodology, Writing – original draft. KC: Conceptualization, Data curation, Writing – review & editing. YZ: Investigation, Writing – review & editing. ZY: Funding acquisition, Investigation, Writing – original draft. DK: Supervision, Writing – review & editing.

## Footnotes

^1^European Community's Rapid Alert System for Food and Feed (RASFF). Available online at: https://webgate.ec.europa.eu/rasff-window/screen/search (accessed 17 January 2024).
