# Supplementary material for: Green control for inhibiting Rhizopus oryzae growth by stress factors in forage grass factory
Source: Front Microbiol. 2024 Aug 5;15:1437799. doi: 10.3389/fmicb.2024.1437799 (PMC11330821; doi:10.3389/fmicb.2024.1437799)
Supplement: Supplementary file 1 [file Data_Sheet_1.docx]

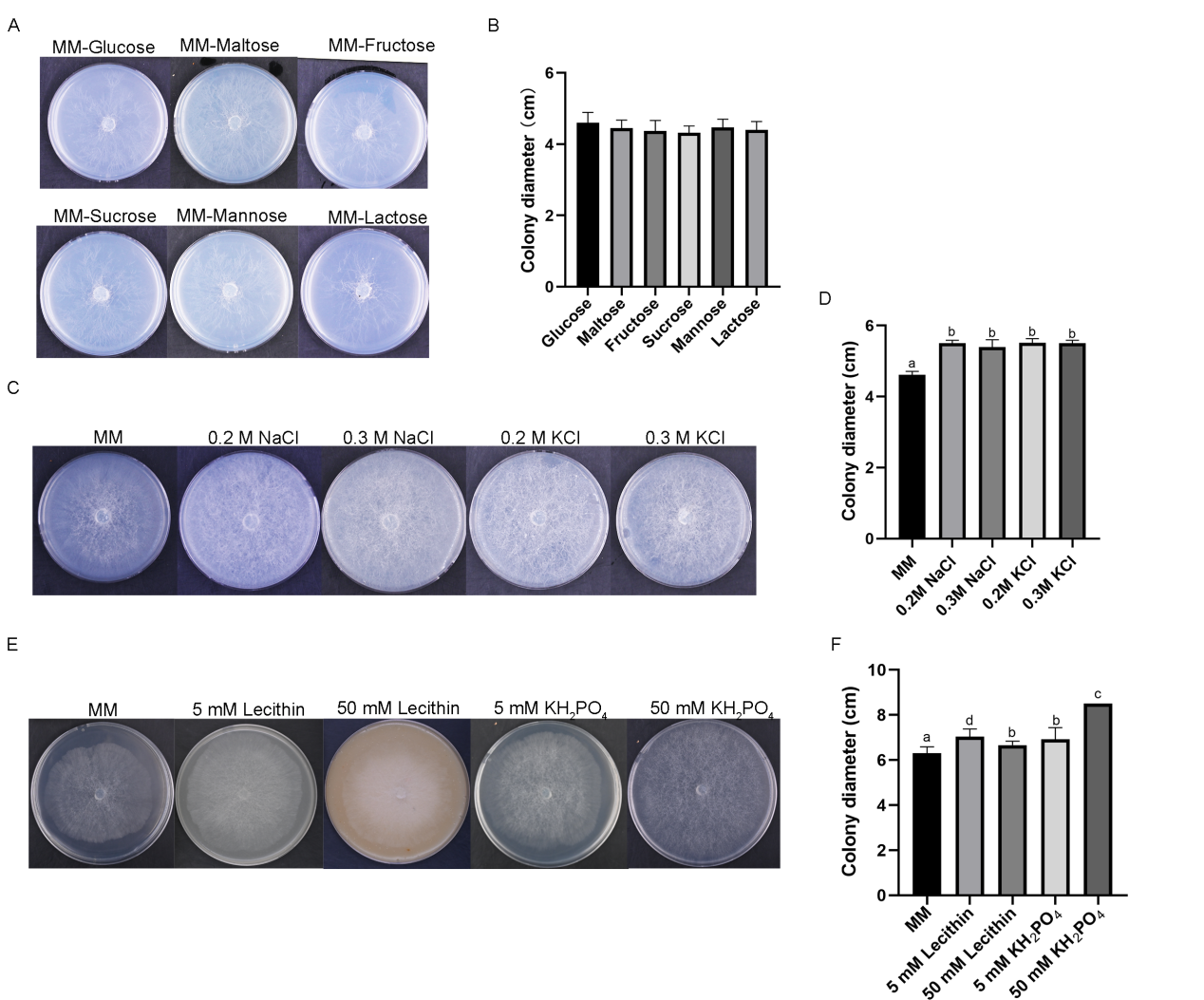


**Figure S1 The effect of different carbon sources, nitrogen sources, salt stress, and phosphorus culture conditions on *R. oryzae*.** (A) The strain cultured on MM media containing maltose, fructose, sucrose, mannose, and lactose under dark conditions for 1 d. (B) The colony diameter on MM media containing maltose, fructose, sucrose, mannose, lactose. (C) The strain cultured on MM media containing 0.2 M sodium chloride, 0.3 M sodium chloride, 0.2 M potassium chloride, and 0.3 M potassium chloride under dark conditions for 1 d. (D) The colony diameter on MM media containing 0.2 M sodium chloride, 0.3 M sodium chloride, 0.2 M potassium chloride, and 0.3 M potassium chloride. (E) The strain cultured on MM media containing 5 mM lecithin, 50 mM lecithin, 5 mM potassium dihydrogen phosphate, and 50 mM potassium dihydrogen phosphate under dark conditions for 1 d. (F) The colony diameter on MM media containing 5 mM lecithin, 50 mM lecithin, 5 mM potassium dihydrogen phosphate, and 50 mM potassium dihydrogen phosphate.


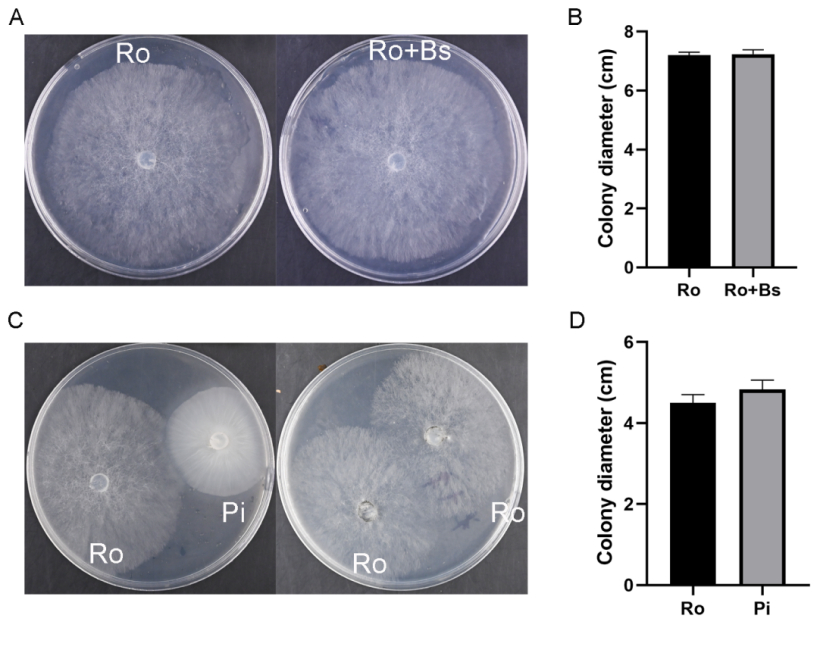


**Figure S2 Effect of *Piriformis indica* and *Bacillus subtilis* on control of *Rhizopus oryzae*.** (A) The dural culture method of *B. subtilis* and *R. oryzae*. Ro represented *R. oryzae*. Bs represented *B. subtilis*. (B) The colony diameter of *R. oryzae* in the dural culture method of *B. subtilis* and *R. oryzae*. (C) The dural culture method of *P. indica* and *R. oryzae*. Ro represented *R. oryzae*. Pi represented *P. indica*. (D) The colony diameter of *R. oryzae* in the dural culture method of *P. indica* and *R. oryzae*.


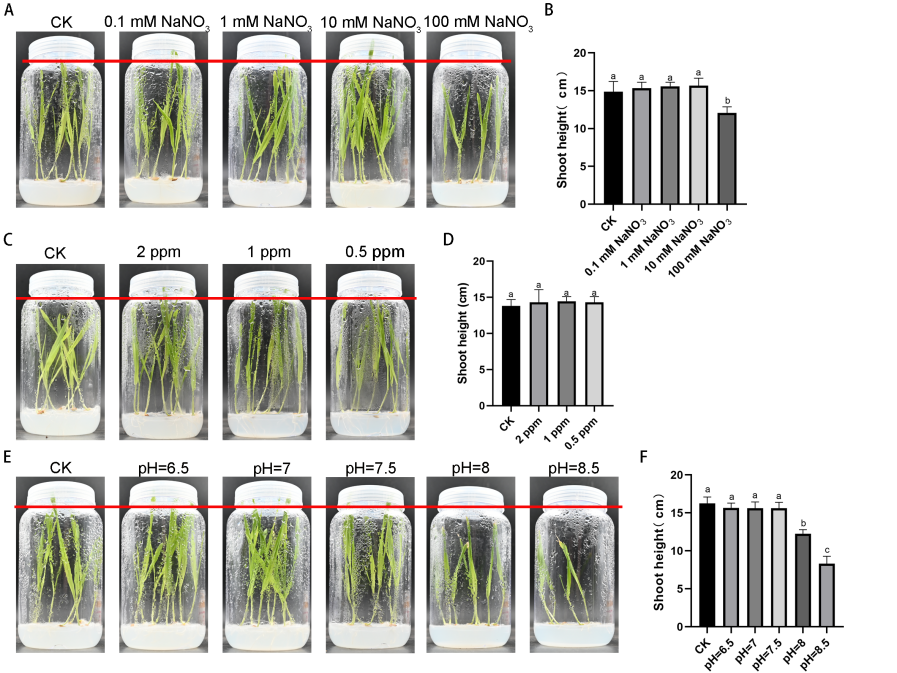


**Figure S3 Growth of barley under different stress factors.** (A) The phenotype of barley grown for 7 d under 0.1 mM NaNO_3_, 1 mM NaNO_3_, 10 mM NaNO_3_, and 100 mM NaNO_3_. (B) The shoot height of barley grown for 7 d under 0.1 mM NaNO_3_, 1 mM NaNO_3_, 10 mM NaNO_3_, and 100 mM NaNO_3_. (C) The phenotype of barley grown for 7 d under 2 ppm ozone water, 1 ppm ozone water, and 0.5 ppm ozone water. (D) The shoot height of barley grown for 7 d under 2 ppm ozone water, 1 ppm ozone water, and 0.5 ppm ozone water. (E) The phenotype of barley grown for 7 d under different pH. (F) The shoot height of barley grown for 7 d under different pH. Error bars mean standard deviation from three replicate samples. Different letters represented significant differences (*p*<0.05).

Table S1 Primers mentioned in the article.

| **Primer name** | **Primer sequence (5'-3')** |
| --- | --- |
| ITS1 | TCCGTAGGTGAACCTGCGG |
| ITS4 | TCCTCCGCTTATTGATATGC |
